# Synthesis and crystal structure of NaCsB_5_O_8_(OH)·H_2_O

**DOI:** 10.1107/S2056989026006018

**Published:** 2026-06-12

**Authors:** Mei-Jun Zou, Xiao-Liang Zhou, Jian-Wen Cheng, Yi-Hang Wen

**Affiliations:** a Key Laboratory of the Ministry of Education for Advanced Catalysis Materials Institute of Physical Chemistry Zhejiang Normal University,Jinhua Zhejiang 321004 People’s Republic of China; Vienna University of Technology, Austria

**Keywords:** alkali metal borates, penta­borate anion, solvothermal synthesis, hydrogen bond, crystal structure

## Abstract

The solvothermal reaction of H_3_BO_3_, Cs_2_CO_3_, NaBO_2_·4H_2_O, formic acid and ethanol led to a new mixed alkali metal borate, NaCsB_5_O_8_(OH)·H_2_O, sodium caesium penta­borate hydroxide monohydrate. Its crystal structure contains penta­borate [B_5_O_10_(OH)]^6–^ building units, and displays a layered structure containing nine-membered rings. The layers extend parallel to the *ab* plane and are stacked along the *c* axis. Na^+^ and Cs^+^ cations are situated between the layers and bond to oxygen atoms with coordination numbers of 7 and 10, respectively. The water mol­ecule likewise occupies the inter­layer space and is hydrogen-bonded to the layers, both as a donor and an acceptor.

## Abstract 2

In the title compound, penta­borate [B_5_O_10_(OH)]^6–^ building units are inter­connected to form (001) layers containing nine-membered rings. Na^+^ and Cs^+^ cations as well as water mol­ecules occupy the inter­layer space.

## 1. Chemical context

Crystalline borates have been well recognized as promising ultraviolet and deep-ultraviolet nonlinear optical and birefringent materials (Zhang *et al.*, 2026 ▸; Li *et al.*, 2023*a* ▸,*b* ▸; Lu *et al.*, 2024 ▸; Ou *et al.*, 2025 ▸; Zou *et al.*, 2026 ▸). Borates exhibit a remarkably rich structural chemistry originating from the various coordination modes of boron to oxygen atoms: BO_3_ triangles and BO_4_ tetra­hedra that can be inter­connected via corner-sharing oxygen atoms to construct a variety of polyborate anions (Lin & Yang, 2011 ▸; Huang *et al.*, 2019 ▸; Chen *et al.*, 2024*b* ▸). To date, more than 3900 borate compounds have been documented in the literature (Mutailipu *et al.*, 2021 ▸).

Penta­borates constitute a family of structurally rich borates, whose fundamental building units typically comprise two B_3_O_3_ rings that are nearly orthogonal to each other. The linkage modes between BO_4_ tetra­hedra and BO_3_ triangles give rise to a series of penta­borate anions, including [B_5_O_10_]^5–^, [B_5_O_11_]^7–^, [B_5_O_12_]^9–^, and [B_5_O_14_]^13–^. Hydroxyl-functionalized penta­borate anions are also well-documented, such as [B_5_O_10_(OH)]^6–^, [B_5_O_8_(OH)_2_]^3–^, [B_5_O_9_(OH)_3_]^6–^ and [B_5_O_6_(OH)_4_]^−^ (Wei *et al.*, 2014 ▸; Ding *et al.*, 2018 ▸). Moreover, extended crystalline frameworks of borates can be formed through condensation reactions accompanied by the elimination of water mol­ecules (Li *et al.*, 2024 ▸; Shi *et al.*, 2019 ▸; Zhao *et al.*, 2022 ▸,2024 ▸; Chen *et al.*, 2024*a* ▸; Wang *et al.*, 2025 ▸; Chen & Yang, 2024 ▸).

In this work, we present the solvothermal synthesis and single-crystal X-ray structure analysis of a novel mixed alkali-metal penta­borate, NaCsB_5_O_8_(OH)·H_2_O, (I).

## 2. Structural commentary

**Figure 1:**
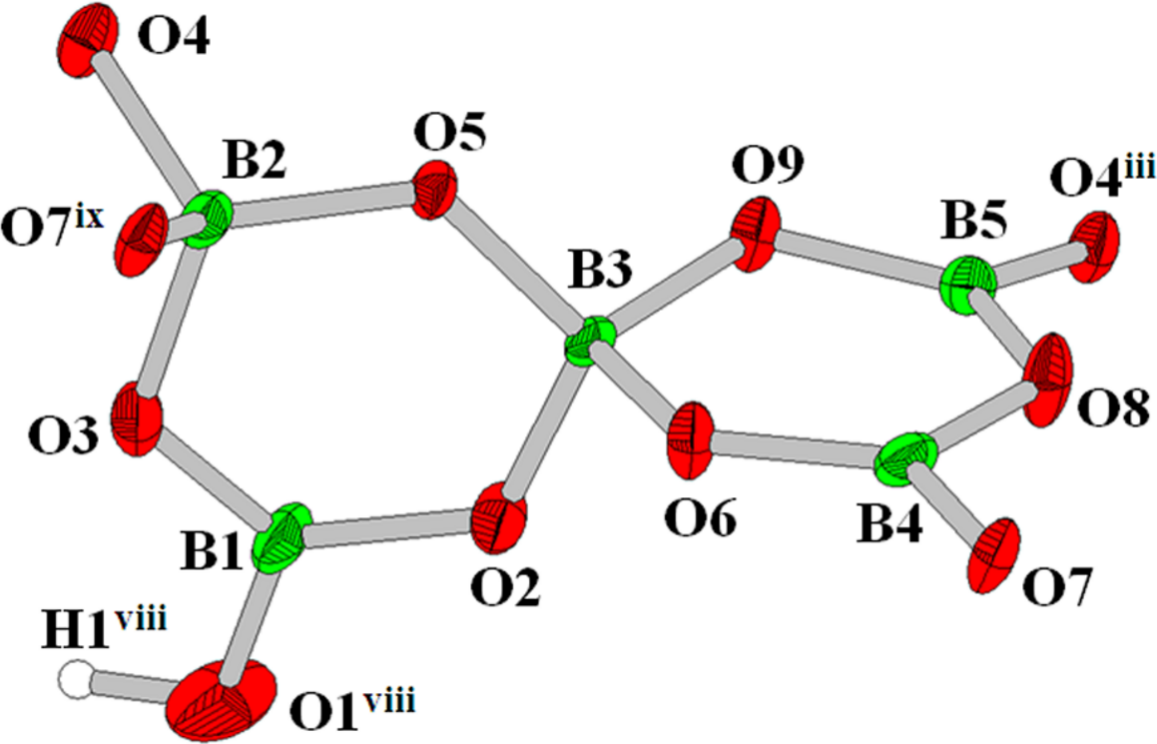
The [B_5_O_10_(OH)]^6–^ building unit in (I) with displacement ellipsoids drawn at the 50% probability level. [Symmetry codes: (iii) −*x* + 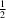, *y* + 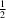, *z*; (viii) −*x* + 1, *y* − 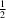, −*z* + 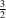; (ix) −*x* − 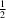, *y* − 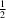, *z*.]

**Figure 2:**
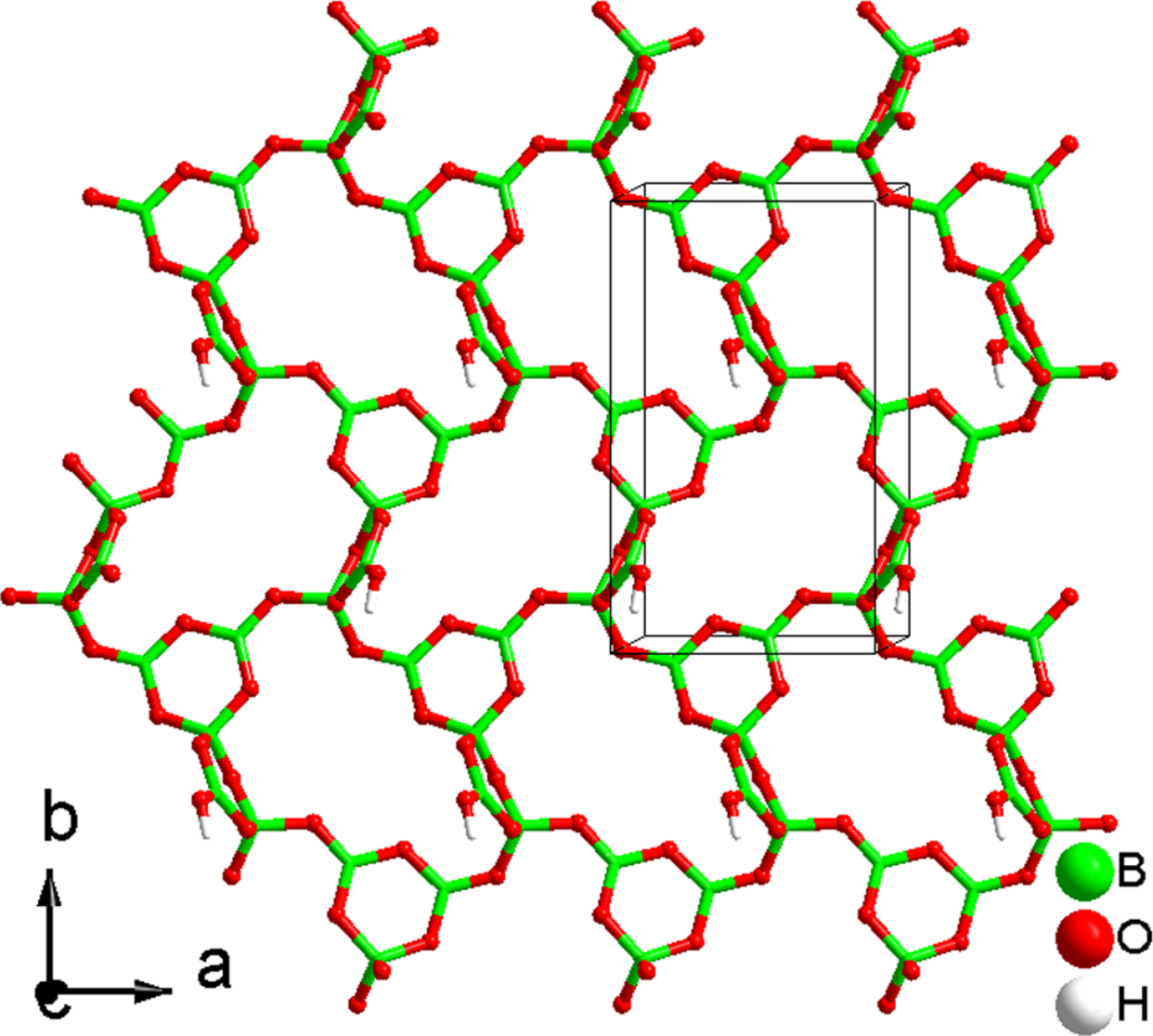
Formation of anionic layers extending parallel to the *ab* plane.

**Figure 3:**
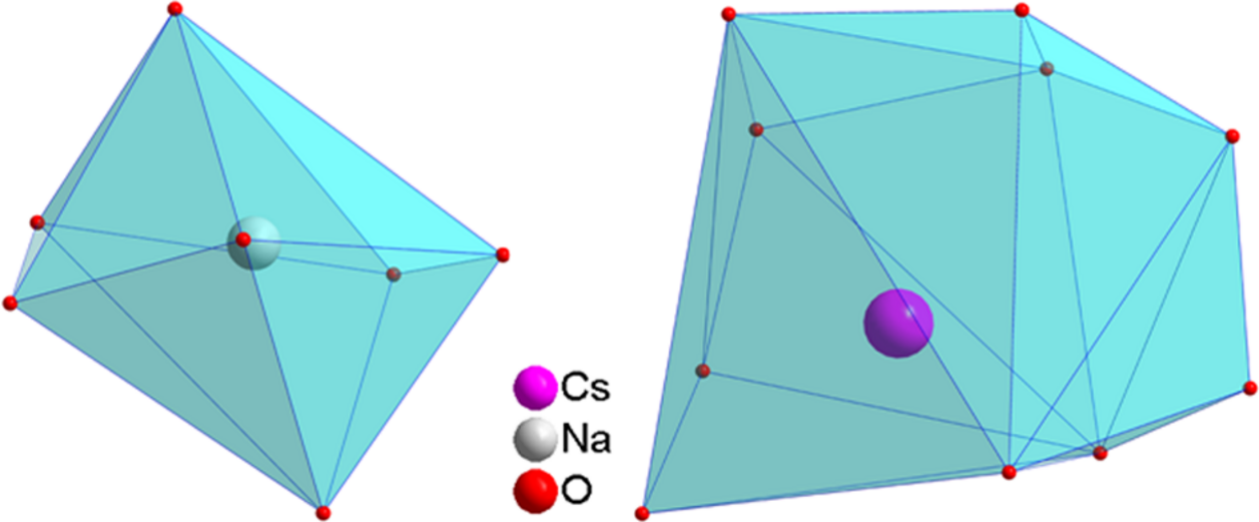
The coordination polyhedra around Na^+^ and Cs^+^ cations.

The asymmetric unit of (I) consists of one formula unit. The penta­borate [B_5_O_10_(OH)]^6–^ building unit (Fig. 1 ▸) is constructed by the linkage of two BO_3_ triangles (B4, B5), two BO_4_ tetra­hedra (B2, B3), and one BO_2_(OH) group (B1), with the B—O bond lengths falling in the range of 1.340 (8) to 1.502 (8) Å (Table 1 ▸). Furthermore, each [B_5_O_10_(OH)]^6–^ unit links to four identical units, giving rise to a layered _∞_^2^[B_5_O_8_(OH)]^2–^ anion extending parallel to the *ab* plane featuring nine-membered rings (Fig. 2 ▸). The layers stack along the *c* axis in an …*ABAB*… alternating fashion. The Na^+^ cations, Cs^+^ cations, and crystal water mol­ecules reside in the inter­layer space. The Na^+^ cation is seven-coordinated in a highly distorted penta­gonal–bipyramidal coordination environment, while the Cs^+^ cation is ten-coordinated in a distorted penta­gonal-prismatic coordination environment (Pinsky & Avnir, 1998 ▸), as shown in Fig. 3 ▸. The Na—O bond lengths range from 2.290 (5) to 2.654 (5) Å and the Cs—O bond lengths from 3.054 (4) to 3.604 (4) Å (Table 1 ▸).

**Figure 4:**
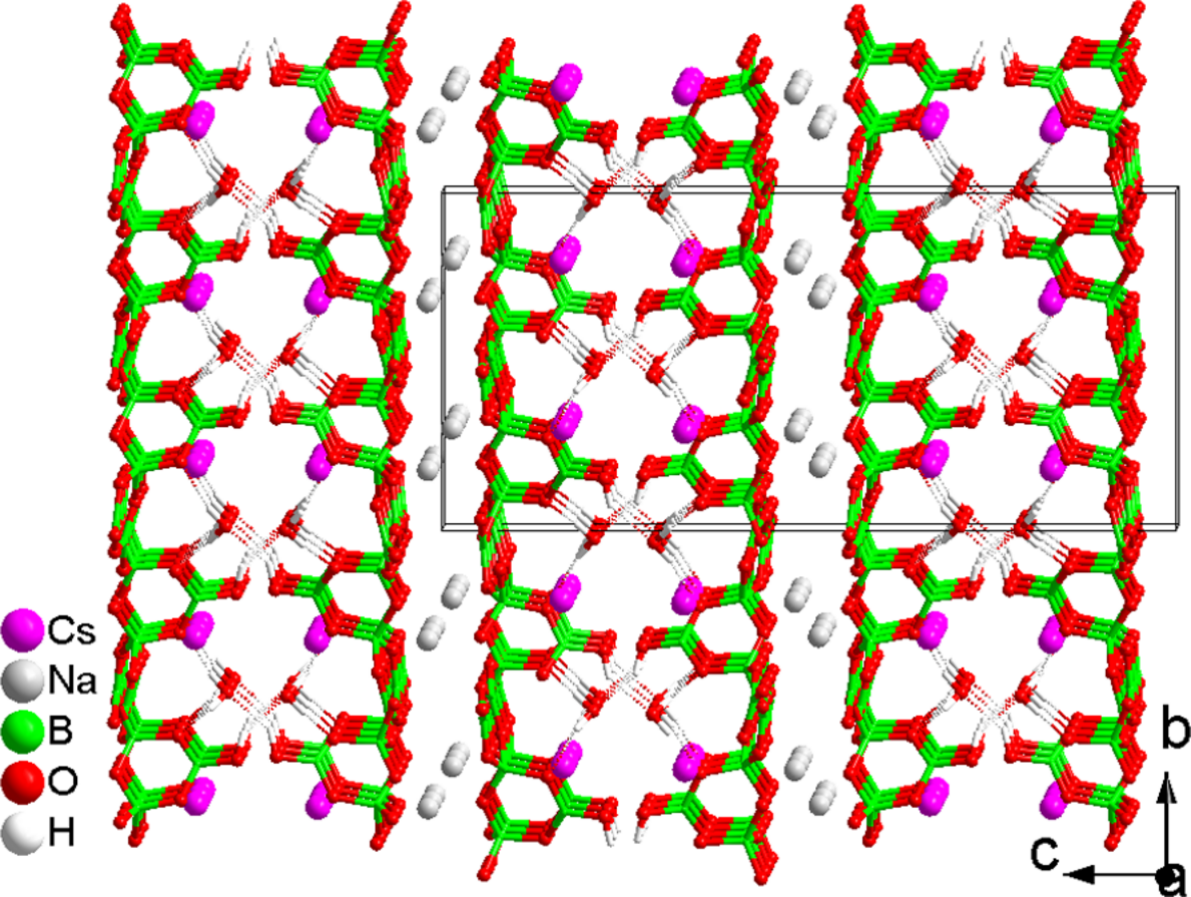
The crystal structure of (I) in a view along the *a* axis. Hydrogen-bonding inter­actions are shown as dashed lines; bonds to the cations are not shown for clarity.

A view of the crystal structure is given in Fig. 4 ▸. The extended structure is consolidated by hydrogen-bonding inter­actions between the hy­droxy group (O1) and the water mol­ecule (O10), and between the water mol­ecule and the anionic framework (Table 2 ▸).

## 3. Database survey

A search of the Inorganic Crystal Structure Database (ICSD, version 5.6.0, updated January 2026; Zagorac *et al.*, 2019 ▸) for alkali metal compounds with the [B_5_O_8_(OH)]^2–^ anion returned seven hits: NaKB_5_O_8_(OH)·H_2_O (triclinic, *P*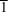; Li *et al.*, 2024 ▸), LiRbB_5_O_8_(OH)·H_2_O (monoclinic, *P*2_1_/*n:* Shi *et al.*, 2019 ▸), K_2_B_5_O_8_(OH)·2H_2_O (ortho­rhom­bic, *Pna*2_1_; Shi *et al.*, 2019 ▸), Rb_2_B_5_O_8_(OH) (ortho­rhom­bic, *Pca*2_1_; Qiu *et al.*, 2021 ▸), LiCsB_5_O_8_(OH)·H_2_O (monoclinic, *P*2_1_/*c*; Chen *et al.*, 2017 ▸), LiKB_5_O_8_(OH)·1.5H_2_O (ortho­rhom­bic, *C*222_1_; Li & Yang, 2019 ▸), and Na_2_B_5_O_8_(OH)·2H_2_O (ortho­rhom­bic, *Pna*2_1_; Corazza *et al.*, 1975 ▸, Wang *et al.*, 2009 ▸). Compared with compound (I), the seven compounds adopt a similar layered structure and share the same [B_5_O_10_(OH)]^6–^ building unit, but they contain different alkali metal ions and possess different space groups, making (I) unique.

## 4. Synthesis and crystallization

A mixture of H_3_BO_3_ (0.3710 g, 6 mmol), Cs_2_CO_3_ (0.3258 g, 1 mmol), NaBO_2_·4H_2_O (0.1378 g, 1 mmol), formic acid (0.25 ml) and ethanol (4 ml) was sealed in a 30 ml Teflon-lined autoclave at 453 K for 6 d and then cooled to room temperature. Colorless crystals of (I) were obtained by filtration, washed with distilled water, and dried in air.

## 5. Refinement

Crystal data, data collection and structure refinement details are summarized in Table 3 ▸. H atoms bonded to O atoms were positioned geometrically and refined using a riding model [O_hydrox­yl_—H = 0.91 Å and O_water_—H = 0.87 Å, *U*_iso_(H) = 1.5 *U*_eq_(O)]. O10 of the water mol­ecule exhibits relatively large displacement parameters, suggesting the presence of potential positional disorder. In the current structure refinement, O10 was modeled as a single site.

## Supplementary Material

Crystal structure: contains datablock(s) I, 1. DOI: 10.1107/S2056989026006018/wm5800sup1.cif

Structure factors: contains datablock(s) I. DOI: 10.1107/S2056989026006018/wm5800Isup2.hkl

CCDC reference: 2545474

Additional supporting information:  crystallographic information; 3D view; checkCIF report

## Figures and Tables

**Table 1:** Selected bond lengths (Å)

Cs—O1^i^	3.110 (5)	B1—O1^viii^	1.382 (9)
Cs—O2	3.054 (4)	B1—O2	1.363 (8)
Cs—O2^ii^	3.604 (4)	B1—O3	1.353 (9)
Cs—O3^ii^	3.406 (5)	B2—O3	1.502 (8)
Cs—O3^iii^	3.335 (4)	B2—O4	1.474 (8)
Cs—O4^iii^	3.324 (4)	B2—O5	1.429 (8)
Cs—O6^ii^	3.313 (4)	B2—O7^ix^	1.493 (8)
Cs—O7^iv^	3.356 (4)	B3—O2	1.478 (8)
Cs—O9	3.111 (4)	B3—O5	1.433 (8)
Cs—O10	3.407 (11)	B3—O6	1.493 (8)
Na—O4^v^	2.652 (5)	B3—O9	1.480 (8)
Na—O5^vi^	2.431 (5)	B4—O6	1.358 (8)
Na—O5	2.290 (5)	B4—O7	1.340 (8)
Na—O6^vi^	2.457 (5)	B4—O8	1.408 (9)
Na—O7^vii^	2.567 (5)	B5—O4^iii^	1.340 (9)
Na—O8^iv^	2.563 (5)	B5—O8	1.399 (8)
Na—O9	2.654 (5)	B5—O9	1.366 (8)

Symmetry codes: (i) 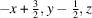; (ii) 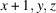; (iii) 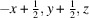; (iv) 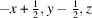; (v) 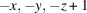; (vi) 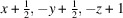; (vii) 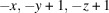; (viii) 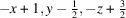; (ix) 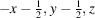.

**Table 2:** Hydrogen-bond geometry (Å, °)

*D*—H⋯*A*	*D*—H	H⋯*A*	*D*⋯*A*	*D*—H⋯*A*
O1—H1⋯O10	0.91	1.90	2.683 (9)	143
O10—H10*A*⋯O3^iii^	0.87	2.35	3.173 (13)	157
O10—H10*B*⋯O2^ii^	0.87	1.95	2.796 (9)	165

Symmetry codes: (ii) 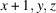; (iii) 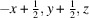.

**Table 3:** Experimental details

Crystal data	
Chemical formula	NaCsB_5_O_8_(OH)·H_2_O
*M*_r_	372.97
Crystal system, space group	Orthorhombic, *Pbca*
Temperature (K)	152
*a*, *b*, *c* (Å)	6.5803 (3), 11.2304 (6), 24.3738 (12)
*V* (Å^3^)	1801.21 (15)
*Z*	8
Radiation type	Mo *K*α
μ (mm^−1^)	4.20
Crystal size (mm)	0.18 × 0.18 × 0.16

Data collection	
Diffractometer	Bruker APEXII CCD
Absorption correction	Multi-scan (*SADABS*; Krause *et al.*, 2015 ▸)
*T*_min_, *T*_max_	0.49, 0.51
No. of measured, independent and observed [*I* > 2σ(*I*)] reflections	21866, 1643, 1228
*R*_int_	0.125
(sin θ/λ)_max_ (Å^−1^)	0.602

Refinement	
*R*[*F*^2^ > 2σ(*F*^2^)], *wR*(*F*^2^), *S*	0.041, 0.091, 1.02
No. of reflections	1643
No. of parameters	154
H-atom treatment	H-atom parameters constrained
Δρ_max_, Δρ_min_ (e Å^−3^)	1.14, −1.24

Computer programs: *APEX2* and *SAINT* (Bruker, 2014 ▸), *SIR2004* (Burla *et al.*, 2007 ▸), *SHELXL* (Sheldrick, 2015 ▸), *OLEX2* (Dolomanov *et al.*, 2009 ▸) and *publCIF* (Westrip, 2010 ▸).

## supplementary crystallographic information

### Sodium caesium pentaborate hydroxide monohydrate Crystal data

**Table:**

NaCsB_5_O_8_(OH)·H_2_O	*D*_x_ = 2.751 Mg m^−^^3^
*M**_r_* = 372.97	Mo *K*α radiation, λ = 0.71073 Å
Orthorhombic, *P**b**c**a*	Cell parameters from 3085 reflections
*a* = 6.5803 (3) Å	θ = 3.3–24.8°
*b* = 11.2304 (6) Å	µ = 4.20 mm^−^^1^
*c* = 24.3738 (12) Å	*T* = 152 K
*V* = 1801.21 (15) Å^3^	Block, colorless
*Z* = 8	0.18 × 0.18 × 0.16 mm
*F*(000) = 1392	

### Sodium caesium pentaborate hydroxide monohydrate Data collection

**Table:**

Bruker APEXII CCD diffractometer	1228 reflections with *I* > 2σ(*I*)
Graphite monochromator	*R*_int_ = 0.125
ω scans	θ_max_ = 25.3°, θ_min_ = 3.3°
Absorption correction: multi-scan (*SADABS*; Krause *et al.*, 2015)	*h* = −7→7
*T*_min_ = 0.49, *T*_max_ = 0.51	*k* = −13→13
21866 measured reflections	*l* = −29→29
1643 independent reflections	

### Sodium caesium pentaborate hydroxide monohydrate Refinement

**Table:**

Refinement on *F*^2^	Primary atom site location: structure-invariant direct methods
Least-squares matrix: full	Hydrogen site location: difference Fourier map
*R*[*F*^2^ > 2σ(*F*^2^)] = 0.041	H-atom parameters constrained
*wR*(*F*^2^) = 0.091	*w* = 1/[σ^2^(*F*_o_^2^) + (0.0194*P*)^2^ + 20.4113*P*] where *P* = (*F*_o_^2^ + 2*F*_c_^2^)/3
*S* = 1.02	(Δ/σ)_max_ = 0.001
1643 reflections	Δρ_max_ = 1.14 e Å^−^^3^
154 parameters	Δρ_min_ = −1.24 e Å^−^^3^
0 restraints	

### Sodium caesium pentaborate hydroxide monohydrate Special details

**Table:**

Geometry. All esds (except the esd in the dihedral angle between two l.s. planes) are estimated using the full covariance matrix. The cell esds are taken into account individually in the estimation of esds in distances, angles and torsion angles; correlations between esds in cell parameters are only used when they are defined by crystal symmetry. An approximate (isotropic) treatment of cell esds is used for estimating esds involving l.s. planes.

### Sodium caesium pentaborate hydroxide monohydrate Fractional atomic coordinates and isotropic or equivalent isotropic displacement parameters (Å^2^)

**Table:**

	*x*	*y*	*z*	*U*_iso_*/*U*_eq_	
Cs	0.54972 (7)	0.30565 (5)	0.66570 (2)	0.03814 (19)	
Na	0.2006 (4)	0.1934 (2)	0.48463 (11)	0.0257 (6)	
B1	0.0294 (12)	0.1797 (6)	0.6701 (3)	0.0197 (16)	
B2	−0.1027 (11)	0.1034 (6)	0.5815 (3)	0.0141 (16)	
B3	0.0647 (11)	0.3085 (6)	0.5883 (3)	0.0129 (14)	
B4	−0.0397 (12)	0.5207 (6)	0.5698 (3)	0.0157 (15)	
B5	0.3167 (11)	0.4635 (6)	0.5607 (3)	0.0150 (16)	
O1	0.9171 (9)	0.6659 (5)	0.7755 (2)	0.0392 (15)	
H1	0.942046	0.588286	0.767268	0.059*	
O2	0.0920 (7)	0.2845 (4)	0.64744 (18)	0.0181 (10)	
O3	−0.0829 (7)	0.0978 (4)	0.64290 (18)	0.0203 (11)	
O4	−0.0143 (6)	−0.0079 (4)	0.56023 (18)	0.0165 (10)	
O5	−0.0124 (6)	0.2079 (4)	0.55865 (16)	0.0123 (9)	
O6	−0.0868 (6)	0.4071 (4)	0.58409 (19)	0.0163 (10)	
O7	−0.1763 (6)	0.6090 (4)	0.56782 (18)	0.0158 (10)	
O8	0.1635 (7)	0.5496 (4)	0.5576 (2)	0.0213 (11)	
O9	0.2635 (7)	0.3464 (4)	0.56586 (18)	0.0158 (10)	
O10	0.946 (2)	0.4776 (7)	0.7082 (3)	0.132 (5)	
H10A	0.875861	0.521456	0.685701	0.198*	
H10B	1.007984	0.426560	0.687130	0.198*	

### Sodium caesium pentaborate hydroxide monohydrate Atomic displacement parameters (Å^2^)

**Table:**

	*U*^11^	*U*^22^	*U*^33^	*U*^12^	*U*^13^	*U*^23^
Cs	0.0248 (3)	0.0548 (4)	0.0349 (3)	0.0060 (3)	−0.0028 (2)	0.0119 (3)
Na	0.0267 (15)	0.0213 (14)	0.0289 (15)	0.0020 (13)	0.0117 (12)	0.0023 (13)
B1	0.029 (4)	0.008 (3)	0.022 (4)	0.002 (3)	−0.002 (4)	−0.002 (3)
B2	0.019 (4)	0.009 (3)	0.015 (4)	0.001 (3)	−0.001 (3)	−0.001 (3)
B3	0.013 (3)	0.008 (3)	0.017 (3)	0.001 (3)	0.005 (3)	−0.003 (3)
B4	0.017 (4)	0.012 (4)	0.018 (4)	0.002 (3)	0.003 (3)	−0.005 (3)
B5	0.019 (4)	0.012 (4)	0.014 (4)	0.002 (3)	0.001 (3)	0.001 (3)
O1	0.065 (4)	0.033 (3)	0.019 (3)	0.015 (3)	−0.017 (3)	−0.004 (2)
O2	0.021 (2)	0.013 (2)	0.020 (2)	−0.0034 (19)	−0.0026 (19)	0.0007 (19)
O3	0.026 (3)	0.015 (2)	0.021 (2)	−0.007 (2)	0.000 (2)	0.003 (2)
O4	0.013 (2)	0.011 (2)	0.026 (2)	0.0023 (18)	−0.002 (2)	−0.0011 (19)
O5	0.013 (2)	0.010 (2)	0.014 (2)	−0.0036 (18)	−0.0017 (17)	−0.0006 (18)
O6	0.010 (2)	0.010 (2)	0.029 (3)	0.0003 (18)	0.0034 (19)	0.003 (2)
O7	0.010 (2)	0.008 (2)	0.029 (3)	−0.0007 (18)	−0.001 (2)	−0.0024 (19)
O8	0.013 (2)	0.009 (2)	0.042 (3)	0.0019 (19)	0.002 (2)	0.005 (2)
O9	0.013 (2)	0.010 (2)	0.024 (2)	−0.0016 (18)	−0.002 (2)	0.0010 (19)
O10	0.268 (15)	0.052 (5)	0.075 (6)	−0.006 (7)	0.057 (8)	−0.033 (5)

### Sodium caesium pentaborate hydroxide monohydrate Geometric parameters (Å, º)

**Table:**

Cs—B1^i^	3.461 (8)	Na—O7^viii^	2.567 (5)
Cs—B5	3.470 (7)	Na—O8^iv^	2.563 (5)
Cs—O1^ii^	3.110 (5)	Na—O9	2.654 (5)
Cs—O2	3.054 (4)	B1—O1^ix^	1.382 (9)
Cs—O2^i^	3.604 (4)	B1—O2	1.363 (8)
Cs—O3^i^	3.406 (5)	B1—O3	1.353 (9)
Cs—O3^iii^	3.335 (4)	B2—O3	1.502 (8)
Cs—O4^iii^	3.324 (4)	B2—O4	1.474 (8)
Cs—O6^i^	3.313 (4)	B2—O5	1.429 (8)
Cs—O7^iv^	3.356 (4)	B2—O7^x^	1.493 (8)
Cs—O9	3.111 (4)	B3—O2	1.478 (8)
Cs—O10	3.407 (11)	B3—O5	1.433 (8)
Cs—H10A	3.2737	B3—O6	1.493 (8)
Cs—H10B	3.3482	B3—O9	1.480 (8)
Na—Na^v^	3.606 (2)	B4—O6	1.358 (8)
Na—Na^vi^	3.606 (2)	B4—O7	1.340 (8)
Na—B2^v^	3.080 (8)	B4—O8	1.408 (9)
Na—B3	2.975 (7)	B5—O4^iii^	1.340 (9)
Na—B3^v^	2.983 (8)	B5—O8	1.399 (8)
Na—O4^vii^	2.652 (5)	B5—O9	1.366 (8)
Na—O5^v^	2.431 (5)	O1—H1	0.9089
Na—O5	2.290 (5)	O10—H10A	0.8700
Na—O6^v^	2.457 (5)	O10—H10B	0.8700
			
O1^ii^—Cs—O2^i^	90.19 (13)	O5—B2—O3	112.8 (5)
O1^ii^—Cs—O3^iii^	129.45 (12)	O5—B2—O4	113.3 (5)
O1^ii^—Cs—O3^i^	75.20 (13)	O5—B2—O7^x^	106.4 (5)
O1^ii^—Cs—O4^iii^	171.26 (12)	O7^x^—B2—O3	108.0 (5)
O1^ii^—Cs—O6^i^	129.75 (14)	O2—B3—O6	106.4 (5)
O1^ii^—Cs—O7^iv^	105.19 (12)	O2—B3—O9	107.8 (5)
O1^ii^—Cs—O9	142.17 (14)	O5—B3—O2	113.0 (5)
O1^ii^—Cs—O10	88.33 (19)	O5—B3—O6	108.3 (5)
O2—Cs—O1^ii^	98.94 (14)	O5—B3—O9	110.7 (5)
O2—Cs—O2^i^	162.45 (15)	O9—B3—O6	110.6 (5)
O2—Cs—O3^iii^	96.71 (11)	O6—B4—O8	119.2 (6)
O2—Cs—O3^i^	128.53 (11)	O7—B4—O6	123.4 (6)
O2—Cs—O4^iii^	82.37 (11)	O7—B4—O8	117.4 (6)
O2—Cs—O6^i^	130.64 (11)	O4^iii^—B5—O8	122.2 (6)
O2—Cs—O7^iv^	95.17 (11)	O4^iii^—B5—O9	118.7 (6)
O2—Cs—O9	45.61 (12)	O9—B5—O8	119.1 (6)
O2—Cs—O10	147.53 (19)	B1^xi^—O1—Cs^xii^	138.7 (4)
O3^i^—Cs—O2^i^	39.82 (10)	B1^xi^—O1—Cs^xi^	78.1 (4)
O3^iii^—Cs—O2^i^	88.83 (11)	B1^xi^—O1—H1	105.9
O3^iii^—Cs—O3^i^	126.91 (7)	Cs—O2—Cs^xiii^	162.45 (15)
O3^iii^—Cs—O7^iv^	120.85 (11)	B1—O2—Cs^xiii^	73.0 (4)
O3^i^—Cs—O10	83.94 (19)	B1—O2—Cs	107.8 (4)
O3^iii^—Cs—O10	56.14 (19)	B1—O2—B3	121.0 (5)
O4^iii^—Cs—O2^i^	90.89 (10)	B3—O2—Cs	104.4 (3)
O4^iii^—Cs—O3^i^	110.81 (11)	B3—O2—Cs^xiii^	89.3 (3)
O4^iii^—Cs—O3^iii^	41.92 (11)	Cs^iv^—O3—Cs^xiii^	133.91 (14)
O4^iii^—Cs—O7^iv^	83.23 (11)	B1—O3—Cs^xiii^	80.9 (4)
O4^iii^—Cs—O10	86.08 (18)	B1—O3—Cs^iv^	123.5 (4)
O6^i^—Cs—O2^i^	39.98 (10)	B1—O3—B2	120.4 (5)
O6^i^—Cs—O3^iii^	60.94 (11)	B2—O3—Cs^xiii^	94.1 (3)
O6^i^—Cs—O3^i^	68.00 (10)	B2—O3—Cs^iv^	102.3 (3)
O6^i^—Cs—O4^iii^	50.92 (10)	Na^vii^—O4—Cs^iv^	77.98 (12)
O6^i^—Cs—O7^iv^	67.69 (10)	B2—O4—Cs^iv^	103.5 (3)
O6^i^—Cs—O10	55.57 (16)	B2—O4—Na^vii^	129.0 (4)
O7^iv^—Cs—O2^i^	67.85 (10)	B5^iv^—O4—Cs^iv^	84.8 (4)
O7^iv^—Cs—O3^i^	42.00 (11)	B5^iv^—O4—Na^vii^	105.3 (4)
O7^iv^—Cs—O10	113.51 (17)	B5^iv^—O4—B2	125.7 (5)
O9—Cs—O2^i^	120.84 (11)	Na—O5—Na^vi^	99.56 (15)
O9—Cs—O3^iii^	76.38 (11)	B2—O5—Na	120.2 (4)
O9—Cs—O3^i^	113.76 (11)	B2—O5—Na^vi^	102.8 (4)
O9—Cs—O4^iii^	42.26 (11)	B2—O5—B3	126.7 (5)
O9—Cs—O6^i^	85.23 (11)	B3—O5—Na	103.7 (4)
O9—Cs—O7^iv^	72.01 (11)	B3—O5—Na^vi^	97.7 (4)
O9—Cs—O10	128.16 (18)	Na^vi^—O6—Cs^xiii^	80.81 (13)
O4^vii^—Na—O9	152.63 (17)	B3—O6—Cs^xiii^	100.7 (3)
O5—Na—O4^vii^	95.63 (17)	B3—O6—Na^vi^	94.9 (3)
O5^v^—Na—O4^vii^	122.56 (17)	B4—O6—Cs^xiii^	129.9 (4)
O5—Na—O5^v^	141.70 (14)	B4—O6—Na^vi^	112.7 (4)
O5—Na—O6^v^	156.68 (19)	B4—O6—B3	124.2 (5)
O5^v^—Na—O6^v^	58.07 (15)	Na^viii^—O7—Cs^iii^	78.48 (12)
O5—Na—O7^viii^	107.02 (17)	B2^xiv^—O7—Cs^iii^	96.3 (3)
O5^v^—Na—O7^viii^	55.78 (14)	B2^xiv^—O7—Na^viii^	95.0 (3)
O5^v^—Na—O8^iv^	108.50 (17)	B4—O7—Cs^iii^	107.2 (4)
O5—Na—O8^iv^	73.26 (16)	B4—O7—Na^viii^	134.4 (4)
O5—Na—O9	57.44 (15)	B4—O7—B2^xiv^	127.9 (5)
O5^v^—Na—O9	84.67 (16)	B4—O8—Na^iii^	128.6 (4)
O6^v^—Na—O4^vii^	67.76 (15)	B5—O8—Na^iii^	102.8 (4)
O6^v^—Na—O7^viii^	95.33 (16)	B5—O8—B4	120.9 (5)
O6^v^—Na—O8^iv^	89.14 (17)	Na—O9—Cs	125.63 (16)
O6^v^—Na—O9	135.81 (18)	B3—O9—Cs	101.8 (3)
O7^viii^—Na—O4^vii^	116.43 (17)	B3—O9—Na	87.2 (3)
O7^viii^—Na—O9	79.70 (15)	B5—O9—Cs	93.3 (4)
O8^iv^—Na—O4^vii^	87.28 (16)	B5—O9—Na	126.5 (4)
O8^iv^—Na—O7^viii^	155.80 (18)	B5—O9—B3	122.5 (5)
O8^iv^—Na—O9	80.57 (16)	Cs—O10—Cs^xv^	95.6 (2)
O2—B1—O1^ix^	114.1 (6)	Cs^xv^—O10—H10A	156.6
O3—B1—O1^ix^	122.2 (6)	Cs—O10—H10A	73.9
O3—B1—O2	123.6 (6)	Cs—O10—H10B	78.8
O4—B2—O3	106.3 (5)	Cs^xv^—O10—H10B	93.4
O4—B2—O7^x^	109.9 (5)	H10A—O10—H10B	104.5

Symmetry codes: (i) *x*+1, *y*, *z*; (ii) −*x*+3/2, *y*−1/2, *z*; (iii) −*x*+1/2, *y*+1/2, *z*; (iv) −*x*+1/2, *y*−1/2, *z*; (v) *x*+1/2, −*y*+1/2, −*z*+1; (vi) *x*−1/2, −*y*+1/2, −*z*+1; (vii) −*x*, −*y*, −*z*+1; (viii) −*x*, −*y*+1, −*z*+1; (ix) −*x*+1, *y*−1/2, −*z*+3/2; (x) −*x*−1/2, *y*−1/2, *z*; (xi) −*x*+1, *y*+1/2, −*z*+3/2; (xii) −*x*+3/2, *y*+1/2, *z*; (xiii) *x*−1, *y*, *z*; (xiv) −*x*−1/2, *y*+1/2, *z*; (xv) *x*+1/2, *y*, −*z*+3/2.

### Sodium caesium pentaborate hydroxide monohydrate Hydrogen-bond geometry (Å, º)

**Table:**

*D*—H···*A*	*D*—H	H···*A*	*D*···*A*	*D*—H···*A*
O1—H1···O10	0.91	1.90	2.683 (9)	143
O10—H10*A*···O3^iii^	0.87	2.35	3.173 (13)	157
O10—H10*B*···O2^i^	0.87	1.95	2.796 (9)	165

Symmetry codes: (i) *x*+1, *y*, *z*; (iii) −*x*+1/2, *y*+1/2, *z*.
